# Supplementary material for: Are carbon emissions trading and green financial instruments synergistic? -Comprehensive quantitative research based on content analysis
Source: PLoS One. 2024 Mar 7;19(3):e0298601. doi: 10.1371/journal.pone.0298601 (PMC10919601; doi:10.1371/journal.pone.0298601)
Supplement: S1 Data — (ZIP) [file pone.0298601.s001.zip › Data/地级市Z_F工作报告.zip!/─┌├╔╣┼/░n═╖╩╨/└┤╫╘╣1⁄2╓┌║┼í╛┬φ┐╦╩2╛▌═°í┐_2016.docx]

各位代表：

现在，我代表市人民政府向大会报告工作，请连同《包头市国民经济和社会发展第十三个五年规划纲要（草案）》一并审议，请市政协委员和列席会议的同志提出意见。

一、2015年和“十二五”时期工作回顾

2015年是我市发展历程中极不平凡的一年。面对严峻复杂的经济形势和艰巨繁重的改革发展稳定任务，全市上下认真贯彻落实自治区和市委的决策部署，凝心聚力，攻坚克难，圆满完成报请市人大常委会审议调整后的主要目标任务。预计全年地区生产总值增长8%，固定资产投资增长15%，社会消费品零售总额增长8%，一般公共预算收入增长7.7%，城乡居民人均收入分别增长8%和9%。主要经济指标增速好于全国、全区平均水平。

（一）经济调控取得新成效。着力稳定企业生产经营，在自治区率先出台促进小微企业加快发展等一系列措施，促进工业经济提质增效。全年为工业企业争取各类扶持资金17.2亿元，帮助规模以上73户停产半停产企业恢复了生产，71户企业扭亏增盈。预计规模以上工业增加值达到1200亿元，增长10%，增速位居自治区前列。全年组织实施亿元以上重点项目733个，完成投资2190亿元，增长39.7%。竣工267个，核电燃料元件生产线等一批重点项目建成投产。加大招商引资力度，区外（国内）到位资金658亿元，增长28.1%。下大力气破解融资瓶颈，全市中小微企业贷款余额达到667亿元，增长11.8%。包钢股份、东宝生物成功定向增发，募集资金301.5亿元，金海新能源、泰宏生态等4家企业实现新三板挂牌。市级融资平台累计筹集建设资金468亿元，设立了总规模40亿元的全国首支地市级“美丽乡村”产业基金。

（二）结构调整迈出新步伐。加快工业转型升级，275个亿元以上工业重点项目完成投资924亿元，优质钢、特种钢比重达到80%，电解铝就地转化率达到70%，稀土新材料、高端装备制造、风光新能源等战略性新兴产业占全市规模以上工业的比重达到17.5%。促进服务业增量提质，29个物流重点项目完成投资42.9亿元，公路港、铁路现代综合物流园区建设进展顺利，全市物流业总收入达到540亿元。五当召、美岱召等旅游景区提档升级，旅游总收入实现331亿元，增长25.7%。金融机构贷款余额达到2072亿元、增长12.4%，表外业务及债务融资余额达到881亿元、增长18.8%。全市注册电商企业达到626家，稀土交易所交易额达到240亿元。加快发展现代农牧业，4个农牧业示范园区完成基础设施投资4亿元，入驻企业85家，3个食品加工园区入驻企业37家，全市规模以上农牧业龙头企业达到149家。成功举办第二届中国（国际）牛羊肉产业大会暨首届绿色农畜产品博览会，签约金额超过150亿元。

（三）改革开放增添新动力。大力简政放权，市本级行政权力由6401项精简到3047项。商事登记“三证合一”、“一照一码”全面推行，市场主体新增3.8万户，增长32.5%。深化国企国资改革，一批混合所有制经济项目取得了重大进展。创新科技体制机制，组建包头市科技创新投资有限公司，中科院包头稀土研发中心、上海交大包头材料研究院挂牌成立。“白云鄂博稀土资源研究与综合利用”、“包头特种车辆及其传动系统智能制造”实验室晋升为国家重点实验室，实现自治区零的突破。加快农村牧区改革，建立农牧业龙头企业与农牧民利益联结机制，完成土地、林地确权登记颁证和草原确权承包试点工作。开展生态文明制度改革，实施排污总量前置审批和污染物排放许可证管理，在自治区率先开展碳排放权交易试点工作。推进社会事业改革发展，取消高中择校生招生、实施蒙中医院医联体试点等工作积极推进。创新对外开放机制，满都拉口岸获批常年通关，成功举办第十一届中俄蒙工商论坛，签约项目协议资金40亿元。其他各领域改革均取得阶段性成果。

（四）城乡建设展示新面貌。新都市区幼儿园、地下综合管廊一期等公共服务设施和基础设施陆续建成。北梁腾空区建设全面启动，转龙藏公园、北梁古民居保护性修缮等项目开始实施。打通城区断头路33条，改造城市管网155公里，新建改造公交线路6条，百里公交专线、百里绿道全部建成，环城铁路改造、国际口岸机场等项目开工建设。新建续建市民公园、南郊公园等14个公园广场，治理河道17.2公里，完成景观蓄水5.1公里。固阳至武川、固阳西绕城、山北地区旅游公路等工程开工建设，百灵庙至白云、固阳至红泥井等一批公路建成通车。建设通村油路483公里，发展节水灌溉面积15万亩，增加农业灌溉面积1.4万亩。

（五）环境质量实现新提升。加快实施重点生态工程，林业生态建设完成投资21亿元，造林面积55.3万亩。落实草原生态补奖机制，围封禁牧成果得到巩固。深入开展大气污染治理，投资25亿元，完成大气污染综合治理项目28个，整治燃煤锅炉、窑炉1107台，淘汰“黄标车”5772台，全市空气良好以上天数同比增加57天，群众满意度达到90%。加强黄河水流域污染治理和水源地保护。投资近16亿元，完成稀土废水资源化回用等16项重点工程。加强工业节能降耗，实施包钢焦炉煤气综合利用、烧结余热发电等节能环保项目，提高工业固体废物和工业污水循环利用水平。单位生产总值能耗下降4.5%，超额完成自治区下达的任务。

（六）人民生活得到新改善。财政民生支出达到330亿元，增长13.1%，为民兴办的十项民生工程全部兑现。发放创业担保贷款3.6亿元，创业带动就业2.85万人。全年城镇新增就业4.2万人，大学生就业2万人，农村牧区劳动力转移就业近24万人。养老、医疗、低保和社会救助标准进一步提高，包钢、包铝和一机职工的医疗、生育保险纳入市级统筹。城区直饮水和农村牧区自来水基本实现全覆盖。农畜产品、粮油、城乡水质等检测机构建成运行。投入各类扶贫资金9亿元，实施扶贫项目420个，3.5万贫困人口实现稳定脱贫。开工建设保障性住房3.21万套，建成1.83万套，完成100万平方米旧小区综合改造。倍受关注的北梁棚户区改造，三年累计投资234亿元，征拆安置任务全部完成，12.4万群众圆了宜居梦。实施农村牧区“十个全覆盖”工程，两年累计投资54.2亿元，完成了592个村庄的建设任务。

（七）社会事业呈现新景象。投入9亿元实施了公办幼儿园、职教园区二期建设等工程，新建加固校舍1.75万平方米，新建改建幼儿园37所，青山区等6个旗县区通过了国家县域义务教育资源均衡发展验收。一批重点文化产业项目建成并投入使用，四级公共文化服务网络覆盖率达到81%，文化产业增加值占地区生产总值的比重达到2.1%。公共卫生服务保障水平不断提高，调整优化医疗布局，专科医院建设全面启动。抓好自治区足球改革试点工作，国家（北方）青少年足球夏令营活动基地一期工程建成投入使用。妇女、儿童、老龄、残疾人等各项事业都取得了新的进步。

（八）民主法治建设跨上新台阶。深入推进依法行政，健全完善重大行政执法决定法制审核制度，开展了行政执法全过程记录试点工作，在各级政府普遍建立了法律顾问制度。狠抓安全生产，强化社会治安综合治理，完善公共安全基础设施，“平安包头”建设扎实推进。民族团结进步工作走在全国前列。市政府自觉接受市人大的法律监督、工作监督和市政协的民主监督，认真听取各方各界意见，科学和民主决策水平进一步提升。严格落实党风廉政建设责任制和中央八项规定，认真开展“三严三实”专题教育，着力正风肃纪，干部作风明显转变，干事创业的热情空前高涨。

各位代表，2015年，我们实现了“十二五”胜利收官，为开启“十三五”发展新征程奠定了坚实基础。回顾过去五年的工作，在市委的坚强领导下，全市各族人民守望相助、团结奋斗，战胜各种风险挑战，包头的发展取得了令人瞩目的成就。

——这五年，是包头经济实力明显增强的五年。地区生产总值从2293亿元增加到3700亿元，年均增长10.7%，人均生产总值超过2万美元。一般公共预算收入从139亿元增加到252亿元，年均增长12.6%。累计完成固定资产投资9813亿元，是“十一五”的2.6倍；累计建成亿元以上重大项目1424个，为全市经济社会发展注入了强劲动力。激发全社会经济活力，全市非公经济占比由60.5%提高到68%。五年中，包头经济发展换挡不失速，连续迈上新台阶。

——这五年，是包头产业结构全面优化的五年。三次产业比例由2010年的2.9:51.6:45.5调整为2015年的2.8:48.5:48.7，第三产业占比首次超过第二产业。“东铝、西钢、南高、北装”的工业布局全面构建，传统产业加快改造提升，战略性新兴产业持续壮大。现代物流业“六园区、一支点”布局已经形成，商贸集聚区和专业市场建设成效显著。旅游、金融、电子商务等现代服务业蓬勃发展。现代农牧业发展迈出新步伐，国家级龙头企业、驰名商标和知名品牌数量位居全区前列。

——这五年，是包头城乡环境显著改善的五年。城区基础设施累计完成投资435亿元，新增城区道路面积593万平方米，全市人均公园绿地面积由10.8平方米提高到13平方米。公路建设累计完成投资147亿元，新建改建干线公路800公里，基本实现旗县区通一级公路，行政村油路通畅率由53%提高到90%。开展重点行业和区域环境综合整治，特别是仅用两年时间重点解决了公路铁路沿线和城市出入口的脏乱差问题。全市森林覆盖率由15.1%提高到16.9%，草原植被盖度由29%提高到32%。

——这五年，是包头百姓得到实惠最多的五年。城乡居民人均收入分别达到38340元和13860元，年均增长10.6%和11.9%。城镇新增就业20.3万人，创业带动就业9.5万人。企业退休人员月人均养老金由1506元提高到2398元，城市低保标准由每月380元提高到580元，农村低保标准由每年1800元提高到5044元。为2002名低保家庭大学生提供就学补助，向30多万户低收入农牧民发放了“一吨暖心煤”。推进扶贫攻坚，解决了12万贫困人口的脱贫问题。

——这五年，是包头城市知名度不断提升的五年。我市先后进入全国新型城镇化建设试点城市、国家工业绿色转型发展试点城市、国家稀土产业转型升级试点城市、全国棚户区改造示范市、节能减排财政政策综合示范城市、国家循环经济示范市等10多个试点城市行列，获得国家可持续发展实验区、国家生态文明先行示范区、创建幸福家庭示范市、全国质量魅力城市、全国文明城市“四连冠”等荣誉称号，实现自治区双拥模范城八连冠和全国双拥模范城七连冠。包头的发展已经跃升到更高层次，站到了新的历史起点上。

五年的政府工作实践，使我们深刻体会到：必须坚持科学发展，始终把发展作为第一要务，主动适应把握引领新常态，依靠发展解决前进中的困难和问题；必须全面深化改革，破除体制机制障碍，不断激发经济社会发展的动力和活力；必须扩大对外开放，主动融入国家发展战略，敏锐捕捉新的发展机遇；必须坚持以人为本，高度关注民生，让发展成果由人民共享；必须强化法治保障，规范社会行为，保持社会和谐稳定；特别是必须坚持党的领导，坚决贯彻落实市委的决策部署，充分调动全市上下的积极性和创造性，形成推动发展的强大合力。这是过去发展最根本的经验，也是未来发展最根本的保证。

各位代表，过去的五年，包头发展取得的成绩来之不易。此时此刻，我们不会忘记国家、自治区从发展战略到政策资金给包头的指导和帮助，不会忘记市委十一届五次全委会议以来从思路指引到推动实践给包头带来的巨大变化，不会忘记各位人大代表和政协委员参政议政、谏言履职的倾心努力，更不会忘记广大干部群众为建设幸福美好家园的辛勤付出。在此，我代表市人民政府，向全市各族人民，向人大代表、政协委员，向各民主党派、人民团体，向离退休老领导、老同志，向驻包部队、武警官兵和公安干警，向所有关心支持包头发展的朋友们，表示衷心的感谢和崇高的敬意！

在看到成绩的同时，我们也清醒地认识到，当前我市经济社会发展依然面临不少困难和问题。诸如部分行业产能过剩，一些企业生产经营困难；产业转型升级任务艰巨，创新驱动能力不强；城乡发展不够协调，城市治理水平有待进一步提升；环境污染问题仍然突出，雾霾治理刻不容缓；税收增长放缓，民生和社会事业刚性支出加大，财政收支矛盾进一步凸显；部分干部还存在学习不够、效率不高、本领不强等问题。对此，我们将强化目标和问题导向，采取有力措施，认真加以解决。

二、“十三五”时期发展目标和主要任务

“十三五”时期是全面建成小康社会的决胜阶段，我们既面临着挑战，也面临着机遇。世界经济增长乏力，我国经济正处在“三期叠加”阶段，长期积累的结构性矛盾已经显现，经济下行压力加大。我市产业前端化、产品初级化特征明显，市场竞争力不强，对我们形成了严峻考验。但我们也要看到，新一轮科技革命和产业革命正在孕育形成，我国经济发展长期向好的基本面没有变，国家深入推进“一带一路”、西部大开发、呼包银榆区域发展等战略，自治区打造沿黄沿线经济带，推进呼包鄂协同发展，为我市营造了长期利好的政策环境，催生了新的发展机遇。尤为重要的是，经过多年发展，我市在区位交通、产业基础、城市配套、科技创新、人力资源等方面都具有一定的比较优势，政府和社会诚信度高，新的经济增长点不断形成，发展后劲明显增强，发展前景十分广阔。因此，只要我们统一思想，坚定信心，发挥优势，抢抓机遇，充分调动各方面的积极因素，就一定能够化解矛盾，战胜困难，不断开创发展新局面。

根据市委十一届八次全委会议精神，我市经济社会发展的指导思想是：全面贯彻党的十八大，十八届三中、四中、五中全会精神和中央、自治区、市委的重大决策部署，深入落实习近平总书记系列重要讲话精神，坚持“四个全面”战略布局，坚持创新、协调、绿色、开放、共享发展理念，以提高发展质量和效益为中心，加快形成引领经济发展新常态的体制机制和发展方式，统筹推进经济、政治、文化、社会、生态文明建设和党的建设，保持在内蒙古率先发展优势，全面建成更高质量的小康社会，为实现“第二个百年”奋斗目标奠定坚实基础。

战略定位是：建成“六个基地”“五个中心”“两个屏障”“一个支点”。即建成稀土新材料基地、新型冶金基地、清洁能源输出基地、现代装备制造基地、新型煤化工基地、绿色农畜产品精深加工基地；建成区域性创新创业中心、区域性物流中心、区域性新型产融结合中心、区域性消费中心、区域性文化旅游中心；建成北方重要的生态安全屏障和祖国北疆安全稳定屏障；建设自治区向北、向西开放的战略支点。

总体目标是：在提高发展平衡性、包容性、可持续性的基础上，实现“三个中高”“两个翻番”“一个建成”。即，经济保持中高速，增长速度高于全国全区平均水平；产业进入中高端，多元发展多极支撑竞争力较强的现代产业体系基本形成；收入迈向中高位，城乡居民人均收入保持我国西部地区领先、进入全国地级城市前列。到2020年生产总值和城乡居民人均收入比2010年翻一番以上。全面建成更高质量的小康社会。

实现上述目标，我们要完成好七个方面的主要任务。

一是推动率先发展，壮大综合经济实力。坚持发展第一要务，着力扩大有效需求，加快建设现代基础设施网络，积极培育壮大市场主体，全面加强经济调控和治理，持续做大经济总量。到2020年，地区生产总值年均增长8%左右、突破5000亿元，一般公共预算收入年均增长7%左右、突破350亿元，固定资产投资年均增长12%左右、突破4000亿元，综合经济实力进入全国地级城市前列，区域性中心城市作用更加凸显。

二是加快创新发展，提高发展质量效益。加快科技、产业、金融、互联网应用和体制机制创新，强化人才在创新发展中的作用，不断增强内生动力。到2020年，优质钢、特种钢比重达到95%，电解铝就地转化率达到85%，稀土产业增加值年均增长20%以上，装备制造业增加值年均增长12%左右，新能源装机达到1500万千瓦，全社会科技研发经费占地区生产总值的比重达到2.5%；服务业增加值占比达到53%；农畜产品加工转化率达到70%以上；重点领域和关键环节改革取得决定性成果，创新驱动发展格局初步形成。

三是推进协调发展，构筑平衡发展格局。加快城乡一体化发展，完善体制机制，统筹推进中心城区和外五旗县区发展，坚持物质文明与精神文明并重、经济建设和国防建设融合，在协调发展中拓展空间，在加强薄弱领域中增强发展后劲。到2020年，常住人口城镇化率达到85%，户籍人口城镇化率达到70%，文化产业增加值占地区生产总值的比重达到5%，城乡协调发展格局基本形成。

四是坚持绿色发展，建设生态文明包头。深入实施重大生态保护和修复工程，有效控制能源资源消耗，促进产业低碳循环发展，推动形成绿色发展方式和生活方式。确保单位生产总值能耗、主要污染物减排达标。到2020年，全市森林覆盖率达到18.3%，草原植被盖度达到35%以上，生态环境持续改善，让包头青山常在、绿水长流、蓝天永驻。

五是深化开放发展，拓展合作发展空间。深度融入“一带一路”战略，积极参与“中俄蒙经济走廊”建设，加快满都拉口岸、中欧装备制造园区等开放平台建设，推动跨境铁路、公路等基础设施互联互通。主动融入环渤海、呼包银榆经济区，推动呼包鄂协同发展，积极承接发达地区产业转移，提升对内对外开放和区域合作水平。到2020年，基本建立起开放型经济新体制。

六是突出共享发展，提升人民幸福指数。精心组织实施好重点民生工程，全力打好脱贫攻坚战，不断提高居民收入水平，健全就业、社保、教育、医疗卫生等公共服务体系，努力让改革发展成果更多更公平地惠及人民群众。到2020年，城乡居民人均收入年均分别增长8%左右和9%左右、达到56300元和21300元，市级标准贫困人口全部脱贫，社会保险实现法定人员全覆盖，基本公共服务均等化、优质化水平全区领先。

七是促进和谐发展，维护社会安全稳定。坚持守望相助、团结奋斗，创新社会治理体制机制，全面建设法治包头，加强和改进信访工作，有效化解社会矛盾，健全完善公共安全体系，强化国家安全力量和手段建设，切实做好民族宗教工作，全面巩固和发展民族团结、社会稳定、边疆安宁的政治局面，让包头成为各族人民安居乐业的幸福家园。

各位代表，“十三五”的宏伟蓝图催人奋进，我们坚信，在市委的坚强领导下，通过全市各族人民的团结拼搏和不懈努力，这张宏伟蓝图一定能够变为现实，全面建成更高质量小康社会的目标一定能够如期实现！

三、2016年工作安排

今年是全面建成小康社会决胜阶段的开局之年，也是推进供给侧结构性改革的攻坚之年。我市和全国全区一样，面临着经济增速、工业品价格、实体企业盈利、财政收入增幅下降及经济风险发生概率上升的“四降一升”突出矛盾和问题，“三去一降一补”的供给侧结构性改革任务较为繁重，迫切需要我们以更大的决心和力度，做好各项工作。

2016年政府工作的总体要求是：深入贯彻落实市委十一届八次全委会议、全市经济工作暨城市工作会议精神，紧紧围绕自治区“8337”发展思路和市委“6521”战略定位，坚持创新、协调、绿色、开放、共享发展理念，坚持稳中求进工作总基调，以供给侧结构性改革为主线，以提高发展质量效益为中心，着力增加有效供给，不断扩大有效需求，突出抓好重大项目建设，大力推进产业转型升级，统筹城乡区域协调发展，进一步加强改革创新、法治建设和民生保障，推动经济社会平稳健康发展，为“十三五”开好局、起好步。

今年经济社会发展的主要预期目标是：地区生产总值增长8%左右，固定资产投资增长12%左右，社会消费品零售总额增长9%左右，一般公共预算收入增长7.5%左右，城乡常住居民人均收入分别增长8%和9%左右，城镇登记失业率控制在3.9%以内。实现上述目标任务，我们要做好以下七个方面的工作。

（一）推动供给侧结构性改革，促进经济发展稳中提效。坚持稳定经济增长和推进结构性改革有机结合，提高经济发展质量效益，加快率先发展步伐。

千方百计稳定企业生产经营。加强对经济运行的分析研判和监测调度，深入落实国家、自治区及我市稳增长和促进实体经济发展的政策，完善“一企一策”、“一类一策”帮扶措施。强化煤电油运气等生产要素保障，帮助企业解决实际困难。加大产业资金扶持力度，组织银企对接，推动金融机构加强对企业的融资贷款支持。推进企业协作配套，鼓励钢铁、有色、装备制造、电力等企业加强产业联合重组，对新上项目鼓励优先采购本地产品。在重视大型骨干企业发展的同时，加强对中小微企业的扶持力度。引导企业积极应对市场变化，主动调整产品结构，提高产品品质和附加值，扩大市场占有率，提升企业经营效益。

多措并举降低企业成本。大力开展降低实体经济企业成本行动，加大简政放权、服务基层的力度，降低制度性交易成本。落实好个体私营经济、小微企业减税让利等各项税费减免政策，清理规范涉企收费，减轻企业税费负担。疏通金融资产向实体经济传导途径，降低企业财务成本。研究制定具体办法，适时调整工业用地出让价格，采取分期缴纳、参股等方式，降低企业用地成本。认真落实“五险一金”精简归并政策，降低企业用工成本。进一步完善煤电价格联动机制，全面实施蒙西电网输配电价改革，降低企业用电成本。加快工业与生产性服务业融合，降低企业物流成本。

坚决有力化解过剩产能。制定总体实施方案和分类推进办法，摸清底数、建立台账。对长期亏损、扭亏无望的“僵尸企业”，停止财政补贴和各种形式的保护，制定处置政策，建立相关退出机制。优化存量产能，强化对产能过剩、技术落后、环保不达标、安全有隐患企业的治理，引导企业降耗、增效、提档，推动长期亏损的企业兼并重组、技改升级、转移发展，焕发企业活力。在化解产能过剩过程中，尽可能多兼并重组、少破产清算。

着力扩大有效投资。组织实施亿元以上重点项目820个，突出抓好一批自治区成立70周年献礼项目，确保年内完成投资2400亿元以上，保持自治区领先地位。推进包头北特高压电力外送通道工程。加快达茂、石拐、固阳通用机场建设。开工建设满都拉口岸至白云、110国道北绕城、东河至托县3条一级公路和达茂边防公路。确保南绕城公路、希拉穆仁至百灵庙等6条续建公路全部建成通车。启动城市立体综合交通枢纽建设，完成环城铁路改造工程。加快地铁一期项目前期工作，争取年内开工建设。加快特色旅游路网建设，新建改建连接线和环线公路510公里，基本实现旅游景区景点干线全覆盖。加强项目储备库建设，力争储备亿元以上项目1000个，其中10亿元以上项目230个，加大前期工作力度，确保投资持续稳定增长。

增强金融保障能力。围绕建设区域性新型产融结合中心，建立100亿元的产业发展基金，设立新材料、现代装备制造、节能环保等战略性新兴产业子基金，积极发展资产证券化业务、产权股权交易等基于实体经济的新型产业金融。建立完善融资担保体系，推广“履约保险”，鼓励发展“助保贷”等针对中小微企业的金融创新产品。规范发展互联网金融，促进P2P、P2G、股权众筹等新型融资模式落地。推动资本市场发展，对500家企业进行股权交易上市辅导，鼓励企业在境内外挂牌上市，通过股票、债券市场直接融资。重视保险、证券等业务，加强与社保基金、保险基金的合作。进一步完善金融扶持、考核、奖励等优惠政策，鼓励金融机构加大对地方经济社会发展的支持力度。着力规范金融市场秩序，组建金融资产交易公司，为债权、不良资产等金融资产的处置和流通提供平台，坚决打击非法集资，坚决守住不发生系统性和区域性风险的底线。

全面释放消费潜力。围绕建设区域性消费中心，进一步完善促进消费政策，推动服务业用电、用水、用气、用热等与工业同价。引导和满足餐饮、教育、文化、旅游、信息等服务消费需求，举办包头美食文化节，努力培育发展养老、健康等新的消费增长点，拓展绿色消费和农村牧区消费，持续做大消费总量。大力推进低空产业发展，促进低空旅游消费。把推动房地产去库存与棚户区改造、引进高端人才、完善社会服务功能、提供创新创业场所等方面结合，并给予政策支持，拓宽去库存渠道。取消过时的限制性措施，适时出台购房优惠政策。加快发展住房租赁市场，积极推进农民工市民化，把公租房扩大到非户籍人口。

（二）调整优化产业结构，大力提升产业发展层次。推动传统产业新型化、新兴产业规模化、支柱产业多元化，促进产业由中低端向中高端发展，全面构筑发展新优势。

加快工业转型升级。深入落实《中国制造2025》发展战略，围绕工业基地建设，实施亿元以上工业重点项目330个，确保年内完成投资1100亿元，力争战略性新兴产业占规模以上工业增加值比重提高2个百分点。稀土新材料基地方面，深入实施“稀土+”战略，做好国家稀土产业转型升级试点工作，发挥中国北方稀土集团龙头作用，推动稀土资源合理平衡利用，提高就地转化率。扩大稀土永磁、储氢、抛光、催化等功能性材料产业规模，重点支持永磁电机、镍氢动力电池、蓝宝石切片、光谱转化材料等稀土应用产业，延长产业链条，努力在新能源汽车、节能环保等终端应用领域及制定稀土材料标准方面实现新突破。加快建设稀土新材料深加工中心、稀土镁合金、钐钴永磁材料、高性能钕铁硼、铌钪综合利用等总投资112亿元的32个项目，全力打造国家稀土新材料、高端应用技术研发和产业化示范基地。新型冶金基地方面，支持企业技术改造和新产品开发，大力发展稀土钢和航空航天铝材、新型车体等轻量化铝合金产品，实施包钢新体系80万吨镀锌板、明拓承接建设140万吨不锈钢等47个项目，抓好包铝110万吨合金铝、希铝50万吨合金铝和20万吨氟化铝等项目推进，推动优质钢、特种钢比重达到85%以上，电解铝就地转化率达到75%以上。清洁能源输出基地方面，推进能源产业协调发展，打造新型“煤—电—铝—加”、“煤—电—化”等产业链，形成市场竞争的电力价格优势。大力实施千万千瓦级风电基地、百万千瓦级采煤沉陷区太阳能光伏电站等40个新能源项目，推进光伏设施农业项目和光伏畜牧业一体化项目。抓住国家“十三五”期间大力发展核电产业的机遇，建设国家核燃料研发生产中心。现代装备制造基地方面，重点发展新能源汽车、环保、煤化工、电力等新型装备制造业，积极培育一批高端装备整机和零部件产业。深化与一机、北重等大型军工企业合作，积极引进一批军工转民用、战时应急等领域的高端项目。充分发挥网络协同制造平台作用，力争400台数控机床实现联网。大力实施特种车辆、石油装备等总投资193亿元的47个项目，打造更具核心竞争力的装备制造产业集群。新型煤化工基地方面，打造煤化工示范园区，着力推进总投资197亿元的神华煤制烯烃二期、总投资130亿元的神雾集团电石法乙炔化工等项目建设，实施煤焦油深加工、国储能煤制乙二醇等项目，形成煤化工多联产产业体系，促进煤化工规模化、集约化、高端化发展。积极开展石墨烯、氟硅新材料等研发应用，构建新的产业发展基础。加强园区基础建设，山南园区主规划区实现“八通一平”，山北园区建成区实现“五通一平”。鼓励园区发展“飞地”经济，共建统管、税收分成，发挥园区产业集聚效应，全市产业集中度达到80%。

加快发展现代服务业。实施奥特莱斯、九原万达广场等77个亿元以上商贸流通项目，确保年内完成投资260亿元。加快打造电子商务产业园区，加大对“互联网+传统商业”的扶持力度，支持稀土、煤炭、化工等电商交易平台建设，大力发展跨境电商，力争电子商务交易额突破300亿元。办好稀土产业论坛、国际装备制造博览会和绿色农畜产品博览会等大型展会。围绕建设区域性物流中心，组织实施24个物流重点项目，年内完成投资50亿元，建成公路港和铁路港一期工程，推动铁路港投入运营。做好“无水港”搬迁改造和提档升级工作，争取设立B型保税物流中心。实现包头机场国际航空口岸临时开放，增开国内航线，开辟国际航线，力争空港物流园区年内建成投入运营。推进钢铁建材等物流园区集聚发展，打造土右、达茂、固阳3个“互联网+精品农畜产品物流基地”。大力培育第三方物流、连锁配送企业，加快发展公铁联运、陆空联运等运输方式，构建综合立体物流体系，增强物流服务功能和区域辐射带动能力。围绕建设区域性文化旅游中心，设立文化产业发展基金，吸引社会资本进入，加快实施书画艺术品、演艺会展、大青山文化创意影视产业园等一批文化产业项目。扶持文化企业发展，推进文化体育资产整合和市场化运营。统筹演艺会展和节庆旅游活动，办好第26届全国图书交易博览会，组织中国游牧旅游文化节等活动。推进文化与旅游深度融合，加快建设五当召、美岱召2个品牌景区和希拉穆仁、春坤山、莲花山等5个精品景区，打造满都拉、沟门、希拉穆仁等旅游特色小镇。与呼鄂两市旅游资源深度对接，打通连接京津冀、晋陕宁的“草原沙漠”黄金旅游线路。

开展境内黄河旅游，发展沿黄旅游经济带。加大文化旅游宣传力度，打造特色品牌，提高包头知名度和吸引力。

加快发展“高精强”现代农牧业。围绕建设绿色农畜产品精深加工基地，建立农牧业产业发展基金，破解经营主体融资难问题。大力发展特色农牧业，加大调整种养结构力度，推动“菜薯肉乳”四大主导产业提档升级，形成23万亩高标准蔬菜、100万亩马铃薯、500万只肉羊、17万头奶牛生产基地。大力发展设施农业，加大4个农牧业示范园区和蔬菜产业园区基础设施建设力度，年内入园企业达到100家以上。制定针对龙头企业和合作社的扶持政策，扶持壮大小尾羊、鹿王、骑士等原有龙头企业，引进中牧集团、华西希望等国内知名企业。打造优势品牌，以品牌引领推动现代农牧业发展。拓宽加工原料来源渠道，与周边地区形成区域农副产品集散加工地。规模以上农牧业产业化加工企业实现销售收入110亿元，增长10%。做大润恒、内蒙古大宗畜产品电子交易中心等绿色农畜产品交易平台，推进农畜产品物流配送和展示直销，发展农畜产品电子商务。规模以上流通企业交易额达到100亿元以上，增长11%。狠抓农牧业生产及质量安全管理，推行标准化生产，将规模以上经营主体全部纳入质量安全监管范围。

（三）抓好创新驱动引领，着力增强发展核心动力。大力推进以科技创新为引领的全面创新，让创新活力竞相迸发，创新价值充分体现，加快创新发展步伐。

实施科技创新“五大工程”。扩大包头科技创新基金规模，引进和发展天使基金、风险投资基金、成果转化基金，提高企业科研经费投入。抓好企业创新能力提升工程，支持国家重点实验室建设，推动北重集团、北奔重汽、中核北方核燃料元件公司、包头铝业申报国家级企业技术中心或国家工程技术研究中心，创建创新型试点企业20家，培育国家级高新技术企业20家，科技小巨人企业10家。抓好产学研协同创新工程，支持中科院包头稀土研发中心、北大包头创新研究院、上海交大包头材料研究院、浙江大学包头工研院和中国农大包头农科院等新型研发机构建设，积极鼓励本市科研院所创新发展。抓好创新平台建设工程，围绕重点产业和工业园区，建设一批中试基地和产业技术研究院，促进更多科技成果在我市转化落地。设立中国技术交易所包头分中心，进一步完善科技大市场功能。抓好智能制造创新工程，成立包头智能制造科技创新研发中心，开展智能制造试点示范，重点打造一批智能工厂、智能车间。抓好产业创新引导工程，围绕建设六个基地，年内实施20项重大科技项目，加快推进高等级汽车用稀土钢、高品质铝合金、纳米级稀土抛光液、核电站用高温合金管等项目的研发及产业化。制定鼓励科技人员创新的政策，在股权激励、科技成果转化、收入分成、领办企业等方面大胆创新。

培育“大众创业、万众创新”沃土。围绕建设区域性创新创业中心，全面推进人才强市工程，深入实施人才引进计划，壮大创业创新群体。设立政府创业投资引导基金和创业投资基金，规范众筹融资平台建设。扩大创业担保贷款规模，探索推出创业券、创新券，降低创业成本。加快推动各类科研平台向社会开放，为创业创新提供低成本技术支撑。建立市区两级创业创新服务中心，重点建设电子商务创业园、稀土高新区科技创业服务中心等企业孵化器，为创业者提供全要素专业化服务。建设和认定创业园孵化基地75个，入驻企业达到1000户，创业带动就业1万人以上。

全面开展“智慧城市”建设。实施“互联网+新兴产业”、商贸流通等16个项目，培育新的经济增长点。制定大数据产业发展规划，设立发展基金，组建投资运营公司，支持石拐大数据产业基地建设。加大对公安、城管、国土、规划、环保等部门的信息资源整合力度，打造统一的电子政务体系和政务共享应用平台。建设智慧包头大数据体验中心，加快建设无线城市，实施智慧政务、智慧教育、智慧医疗、智慧社区、智慧旅游、智慧管网等应用项目，力争用3年时间建成体系完善、功能先进、高效快捷、便民共享的“智慧包头”。

（四）深化改革扩大开放，积极谋求重点领域突破。下大力气破除制约发展的体制机制障碍，释放发展新活力，推动发展更高层次的开放型经济，加快开放发展步伐。

深化经济体制改革。加快组建国有资本运营公司和投资公司，推进经营性国有资产集中统一监管，提高国有资本配置和运行效率。深化供销体制改革，组建供销集团。加快建立PPP专家库和项目信息库，创新项目收益和分配机制，实施好普拉特餐厨垃圾处理等一批示范项目。大力支持非公经济健康发展，进一步放宽电力、交通、市政公用等领域市场准入，推广特许经营、投资补助等方式，营造良好服务环境。高度重视本土企业发展，加强企业家队伍建设。

深化行政体制改革。全力做好国家、自治区下放行政审批事项承接工作，进一步规范审批要件和审批流程。深化商事登记制度改革，探索建立受理与审批分离模式，加快推进电子营业执照和企业注册全程电子化，加强“一库两平台”信用体系建设，强化事中事后监管，加强标准化建设，创建全国质量强市示范城市。深化财税体制改革，认真落实国地税征管体制改革举措，抓好财政资金开源节流，推行财政支出绩效评价机制，加强国库专项资金的管理，做好政府存量债务置换工作。积极推广政府购买服务，在教育、医疗、养老等领域扩大适用范围。抓好“扩权强县”改革，完善支持试点旗县发展的配套政策。全面启动土地、房屋、林地、草原等不动产统一登记工作。加快推进人事制度改革。

深化农村牧区改革。加快推进户籍制度改革，促进农牧业转移人口和其他常住人口落户城镇。加快农村牧区产权制度改革，建立集体产权交易市场，全面推开土地承包经营权确权登记颁证工作，健全土地、草牧场流转机制，积极开展土地、草牧场承包经营权抵押贷款业务。完善农牧业龙头企业与农牧民利益联结机制，大力培育种养大户、家庭农牧场、专业合作社和产业化龙头企业等新型农牧业经营主体，重点与农牧民建立订单合同、股份合作、价格保护、服务协作和流转聘用等紧密型利益联结模式，充分保障农牧民利益。

深化生态文明制度改革。完善森林生态效益补偿制度，实施第二期国家生态补奖等项目，探索合理利用草原有效途径。推进碳排放权交易体系建设，积极开展碳排放权跨区域合作。完成排污权初始核定工作，全面推行排污权有偿使用和交易，开展环境污染第三方治理。执行最严格的水资源管理制度，落实“三条红线”控制指标，推进水权转换工作。加快园区配套供水管网建设，完成水源置换。建立节地评价制度，把好预审和供地关，加大闲置土地清查力度，提高土地集约利用水平。此外，我们还要加快推进城镇化、社会事业、民主法治等领域改革，确保完成各项改革任务。

积极扩大对外开放。围绕建设自治区向北、向西开放战略支点，深化国际交流合作，加强满都拉口岸保税仓储区建设，口岸过货量力争突破100万吨。举办中蒙商品展洽会，促进中蒙文化交流。加快实施包满三期铁路项目，抓好满都拉至蒙古国赛音山达铁路公路项目前期工作，加快推动呼包银客专、包西客专开工建设。抓好中欧装备制造园区建设，促成一批新项目落户园区。加强外贸出口服务工作，鼓励包钢、一机、北奔等企业加大适销对路产品开发力度，千方百计扩大出口。加强区域经济合作，主动融入呼包银榆经济区，推动呼包鄂协同发展，力争在基础设施互联互通方面取得新进展。鼓励各地区各部门向上争取政策项目，加大招商引资力度，主动承接发达地区产业转移，积极引进发展总部经济，力争引进区外（国内）到位资金700亿元以上，增长10%。

（五）统筹城乡协调发展，加快构建平衡发展格局。坚持以人为本，转变城市工作理念，提高城市治理能力，统筹城乡区域发展，提高新型城镇化水平，走出具有包头特色的城市发展道路，加快协调发展步伐。

加快新型城镇化建设。大力推进新都市区建设，实施城市展示馆、文化艺术馆、博物馆“三馆”建设工程，新建续建30条道路和3座桥梁，完成包九中分校建设任务，完善公共服务设施，加快推进万达广场、保利、碧桂园等品牌商业地产项目，打造城市新亮点。加大北梁腾空区开发建设力度，新建改建外环路等62条道路，实施包一中新校区等一批公共服务设施项目。完善城区交通体系，开工建设110国道改造、沼南大道等51条道路和8座桥梁，完成打通城区断头路三年规划任务。抓好地下综合管廊建设，在国家地下综合管廊试点城市建设方面取得更大突破。重点加大对外五旗县区城镇规划、土地开发、基础设施建设和融资等方面的支持力度，高标准推进萨拉齐、百灵庙、金山等城关镇和满都拉特色口岸城镇建设，实施固阳天然气输配管道等38个项目，增强城镇支撑产业和吸纳就业能力。从2016年起，我们要用三年时间完成城市棚户区改造任务，今年投资90亿元，改造2.87万户。今年还要投入12亿元，完成510万平方米既有建筑节能改造，到明年年底前，完成所有城区老旧小区改造任务。

提高城市治理能力。强化城市规划的严肃性和约束性，启动国民经济和社会发展规划、土地利用总体规划、城市总体规划“三规合一”工作，凸显舒展通透、简约大气、宜居宜业的城市气质。深化城市管理体制改革，构建市区联动、信息共享、覆盖全市的数字化指挥系统。全面推进城市执法体制改革，改进城市管理工作，在食品药品安全、生态环境、安全生产、公共卫生、劳动保障等领域推进综合执法。加强对城乡结合部、城市棚户区、城中村和重点项目区违法建设的管控，严肃查处私搭乱建和破门开店等行为。实施城市环境综合整治提升工程，打造友谊大街等25条景观示范街，开展鞍山道等35条街巷综合治理。大力发展公共交通，增设停车场，强化智能交通管理，着力解决道路拥堵问题。对物业企业实行信用等级考核制度，提升物业服务水平。引进和推广装配式建筑技术，促进建筑业工业化、绿色化发展。我们要进一步巩固全国文明城市、国家卫生城市创建成果，让城市变得更加整洁靓丽。

促进外五旗县区协同发展。在抓好主城区建设的同时，更加注重外五旗县区的发展。推动石拐资源枯竭区人口转移搬迁，加快喜桂图新区建设，加速融入主城区，推动文化旅游和物流业发展。推进土右旗中等城市建设，加快撤旗设市进程，培育新型能源、煤化工、新材料等主导产业。大力支持山北地区发展，出台山北地区协同发展指导意见和产业发展整体规划，鼓励承接产业转移，从项目、资金和生产要素保障等方面给予山北地区差别化政策支持。推进白云达茂边境口岸城市建设，加快一体化进程，推动稀土资源综合利用、风光新能源、现代煤化工等产业发展。加大对固阳的帮扶力度，扶持钢铁、有色、能源等产业发展。

全力实施农村牧区“十个全覆盖”和脱贫攻坚工程。把“十个全覆盖”作为做好农村牧区工作的重要抓手，打好攻坚战。投资61亿元，实施9个旗县区1020个村庄的“十个全覆盖”工程。加强农村牧区环境标准化管理，积极调动农牧民积极性，建立完善长效管理机制，在自治区率先完成所有嘎查村“十个全覆盖”建设任务，率先实现农村牧区基本公共服务均等化，让农村牧区更美丽、农牧民群众生活更殷实。开展精准扶贫、精准脱贫，整合各类扶贫资金10亿元，实施扶贫项目400个以上，年内解决国家扶贫标准下7000人脱贫问题，到2017年使国家扶贫标准下1.3万贫困人口在自治区率先脱贫、固阳区贫县摘帽。要把扶贫与产业发展相结合，扶贫与扶智相结合，让贫困群众生活更有保障、更有尊严。

（六）全面加强生态环境建设，不断开创生态文明建设新局面。坚持走绿色富市、绿色惠民道路，不断拓展生态文明内涵，狠抓生态恢复和环境治理，实现资源节约高效利用，满足各族人民对良好生态环境的期待，加快绿色发展步伐。

狠抓环境污染防治。实施环境污染综合治理项目112个，提升全市环境质量。深化大气污染综合治理，启动燃煤电厂超低排放升级改造工程。推进型煤基地建设，强化型煤推广应用和产品质量监督，探索光伏、风电等清洁能源替代燃煤集中供热模式，努力解决城区居民燃煤散烧问题。采取有效措施减少扬尘污染和露天焚烧，开展远郊旗县区工业企业治理和燃煤锅炉整治。实施水污染综合治理工程，消除主城区黑臭水体。开展土壤污染治理，加强危险废物和放射性废物的安全处置。

全力推进生态建设。围绕建设北方重要的生态安全屏障，扎实推进国土绿化，完成国家重点林业工程40万亩，六大重点区域绿化4万亩，进一步提高公路铁路沿线和城市出入口环境综合治理水平。推动高坡地退耕还林工作，大力发展经济林。加强大青山国家级自然保护区生态修复和湿地保护，加大水土保持治理力度。强化山北矿区生态建设和保护，实施绿色矿区建设工程。开展北梁棚户区地质环境治理和生态恢复。启动实施黄河堤防加固二期工程，构建新的生态景观带。巩固禁牧成果，加强草原生态保护。高标准实施赛汗塔拉城中草原扩容提质、奥林匹克公园功能拓展、万水泉台地公园等17个精品公园广场项目，完成莫尼林带改造工程。

提高资源利用水平。加快发展循环经济，发挥东河铝业园区国家“城市矿产”、白云鄂博矿区矿产资源综合利用和土右旗固废综合利用示范基地的带动作用，加强资源回收利用，力争工业固废综合利用率达到75%。加强高耗能行业和重点企业节能改造和能耗管控，加快实施包钢烧结余热发电等5个重大节能示范项目。建设企业能源监测管理平台，确保万元工业增加值能耗明显下降。推动依法治水、科学用水，全力做好“水”这篇文章。继续推动昆河、四道沙河等5条河道生态修复和水系建设。启动“海绵城市”建设，规划实施雨污分离工程。加快污水处理厂建设，污水日处理总规模达到40万吨以上。实施西南区域污水资源化利用工程，再生水利用率达到35%。

（七）努力增加民生普惠成果，切实提高共建能力和共享水平。以民之所望为施政所向，有效加强公共服务供给，切实保障群众权益，加快共享发展、和谐发展步伐。

完善就业服务体系。健全就业援助机制，开发公益性岗位2300个，制定出台更加便于大学生就业创业的政策，提高高校毕业生就业水平。实施北梁棚改就业创业示范工程，帮助棚改居民就业。新建、改扩建一批基层就业创业服务平台，健全城乡一体化就业创业公共服务体系。制定结构性失业应对预案，妥善安置破产企业职工，落实援企稳岗、社保补贴、减免税费等政策，实施再就业工程，确保就业形势总体稳定，年内新增城镇就业4.2万人以上。加大新型职业农牧民和进城务工农牧民的培训力度，农村牧区富余劳动力转移就业达到23万人以上。

提高居民收入水平。推进机关事业单位建立基本工资正常调整机制，落实旗县以下机关公务员职务与职级并行制度和乡镇工作补贴制度。强化企业收入分配调控，加强国有企业负责人薪酬管理，扩大企业职工工资集体协商覆盖范围。认真执行国家粮食直补、良种补贴和农资综合补贴政策，扩大农牧民在土地、草牧场承包经营权和流转等方面的收益。实施全民参保计划，城乡居民养老保险参保率达到96%以上。整合基本医疗保险制度，试行总额控制付费方式，推进区域内城镇医疗保险异地就医即时结算工作。建立医保、商业保险与医疗救助联动互补模式，确保困难群众看得起病。

提高教育质量。深化教育综合改革，完善师资培训体系，抓好教师继续教育，推进教育信息化。制定出台吸引社会资本兴办教育的优惠政策，建立学前教育成本分担机制。新建续建幼儿园32所，建成包头市中小学综合实践教育中心。大力推进县域义务教育资源均衡发展。推动中高职有效衔接，加快职教园区二期工程建设，促进校企深度融合。办好民族教育，推进民族学校特色教育和标准化建设。启动特殊教育中心项目。扩大教育开放，实施中小学名优教师引进“百川计划”，鼓励开办国际学校，推动与俄蒙互派留学生。关心支持高校发展，鼓励高校与国外联合办学。我们要落实立德树人根本任务，努力办好人民满意的教育。

加强公共文化服务。深化文艺院团改革，打造具有包头特色的品牌文艺院团。圆满完成国家级公共文化服务体系示范区验收任务。实施公共文化服务“普雨工程”，推进市群艺馆等文化设施建设，进一步完善公共文化服务体系。正确处理文化遗产保护、开发利用、传承创新的关系，重点抓好北梁等历史街区和遗迹的保护开发工作。深入开展文化阵地服务、流动服务和远程数字服务，实现全市公共文化服务全覆盖。建立文艺精品创作保障机制，鼓励文艺作品创作。举办“包克图演出季”，办好“鹿城文化艺术节”，推行文化惠民卡，使群众享受到高品位、低票价的文化服务，进一步提高群众文化素养。

着力打造健康包头。抓好公共卫生服务，推进以城市为重点的公立医院改革，开展城乡医疗机构对口支援，完善分级诊疗制度，推进医师多点执业。加快新都市区医院和儿童医院、蒙中医院等特色专科医院建设，支持民营医院发展，加强乡镇卫生院和标准化社区卫生服务中心建设。抓好体育事业发展，开工建设国家（北方）青少年足球夏令营活动基地二期等工程，确保奥林匹克公园乒羽馆、游泳馆、网球中心年内建成。统筹健身设施布局，构建15分钟健身圈。巩固足球改革成果。大力发展学校体育和专业竞技体育。抓好食品药品安全监管，对150户食品药品企业进行实时电子监管，对校园周边、食品生产加工小作坊等重点领域和行业加大专项整治力度，努力创建国家食品安全城市。完善土右旗、九原区、青山区3个食品加工园区，引进龙头企业20家以上。高度重视养老事业，推行“医养结合”新型养老模式。启动民政福利园区和残疾人康复中心建设。全面落实国家人口生育政策，建立健全农村牧区留守儿童和妇女、老人关爱服务体系。

维护社会和谐稳定。围绕建设祖国北疆安全稳定屏障，加强和创新社会治理。建设基础综合管理服务平台，强化城市和农村牧区社区建设。坚持依法化解信访问题。拓宽仲裁服务领域，加大民商事矛盾纠纷解决力度。强化安全生产监管，落实安全生产责任制，抓好重点领域隐患排查和专项整治，坚决防止重特大事故发生。认真落实党的民族宗教政策，促进民族团结、宗教和顺。关心支持老龄事业和老干部工作。采取有效措施支持国防建设和军队改革，巩固双拥共建成果，争创国家军民深度融合试点城市。加强应急管理，提高防灾救灾能力，及时有效应对突发性事件。深入推进“平安包头”建设，抓好公安基层基础建设，全面加强禁毒工作，完善“四级巡控”网格化警务机制，以信息化为引领，打造社会治安防控包头模式，不断提升人民群众的安全感和满意度。

各位代表，站在新的历史起点，我们要全面加强政府自身建设，努力为全市经济社会发展营造良好的服务环境。

我们要加强法治政府建设，自觉接受市人大法律监督、工作监督和市政协民主监督，认真听取各民主党派、工商联、无党派人士和人民团体的意见建议。深入推进依法治市，加强和改进政府法治工作，坚持依法行政，使政府权力在法治的轨道上运行。

我们要加强诚信政府建设，坚持一切从实际出发，做到因地、因时、因事制宜，扎扎实实做好每一项工作，全面推进政务公开，认真兑现向社会和群众作出的承诺，切实提升政府公信力和影响力，推动形成诚信守法的社会环境。

我们要加强创新政府建设，坚持解放思想、与时俱进，敢于担当、善于担当，进一步提升政府效能。不断加强学习、研究政策，切实提升服务发展、服务基层、服务群众的能力和水平，塑造服务优质、运转高效、充满活力的新形象。

我们要加强廉洁政府建设，全面贯彻市委的决策部署，切实提高执行力和落实力。落实党风廉政建设责任制和“一岗双责”责任制，加强制度建设，强化行政监察和审计监督，建设廉洁从政的干部队伍，营造干部清正、政府清廉、政治清明的执政环境。

各位代表，“十三五”发展的序幕已经拉开，我们肩负着光荣而艰巨的历史使命，包头前进中的困难需要我们去克服，包头人民对美好生活的向往激励我们去努力，包头新的历史篇章等待我们去书写。让我们在自治区党委、政府和市委的坚强领导下，紧紧依靠全市各族人民，满怀激情，锐意进取，以优异成绩迎接自治区成立70周年，为全面建成更高质量的小康社会而努力奋斗！
